# Supplementary material for: Definitive engineering strength and fracture toughness of graphene through on-chip nanomechanics
Source: Nat Commun. 2024 Jul 12;15:5863. doi: 10.1038/s41467-024-49426-3 (PMC11245622; doi:10.1038/s41467-024-49426-3)
Supplement: Supplementary file 1 — Supplementary Information [file 41467_2024_49426_MOESM1_ESM.pdf]

Supplementary Information

Definitive engineering strength and fracture toughness of graphene through on-chip nanomechanics

Sahar Jaddi[1], M. Wasil Malik[2†], Bin Wang[2, 3], Nicola M. Pugno[4,5], Yun Zeng[3], Michael Coulombier[1], Jean-Pierre Raskin[2], Thomas Pardoen[1]

[1]Institute of Mechanics, Materials and Civil Engineering, UCLouvain, Belgium
[2]Institute of Information and Communication Technologies, Electronics and Applied Mathematics, UCLouvain, Belgium
[3]School of Physics and Electronics, Hunan University, China
[4] Laboratory for Bioinspired, Bionic, Nano, Meta Materials & Mechanics, Department of Civil, Environmental and Mechanical Engineering, University of Trento, Trento, *Italy[5] School of Engineering and Material Science, Queen Mary University of London, London, United Kingdom*
[6]WEL Research Institute, avenue Pasteur, 6, 1300 Wavre, Belgium

Supplementary Figures

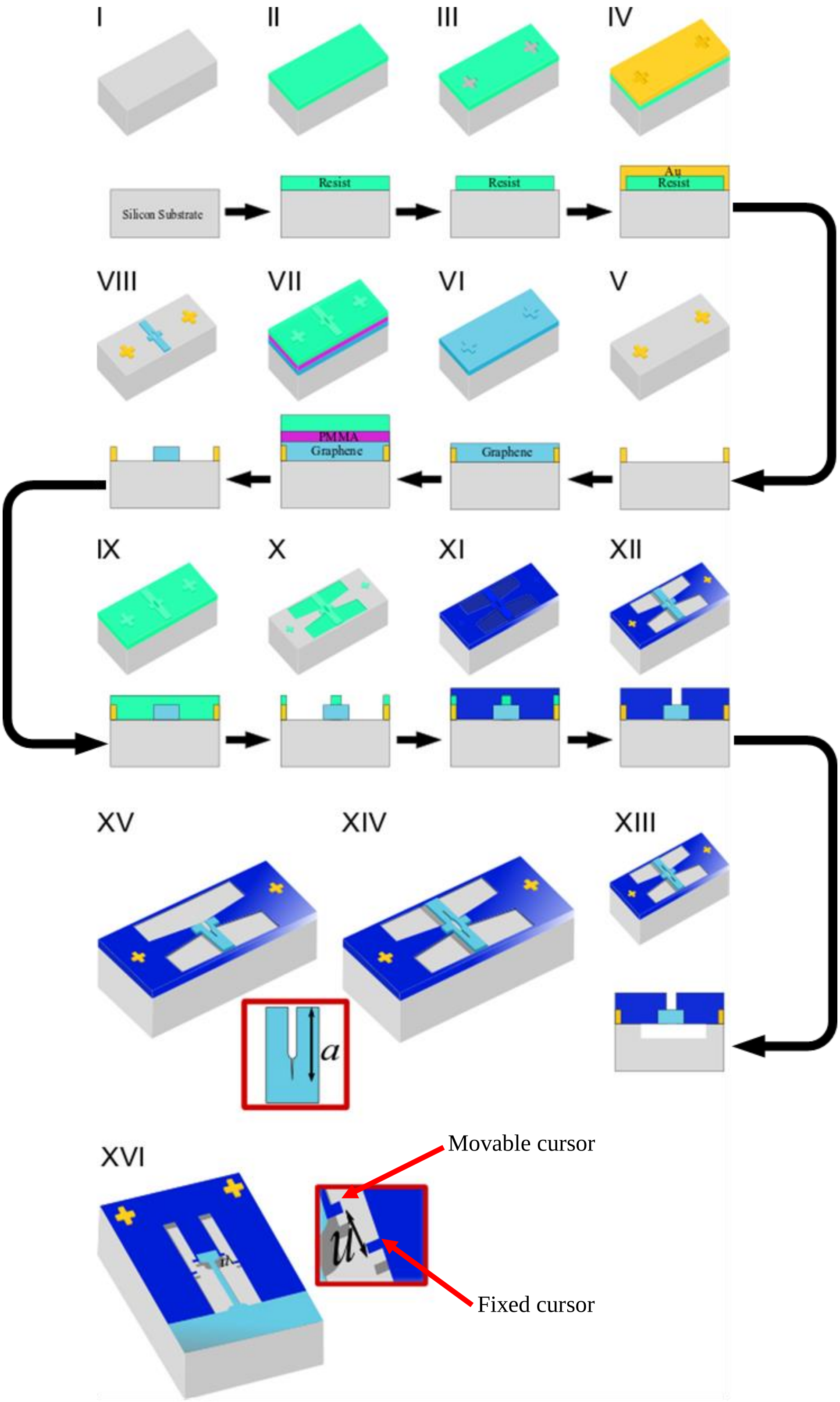

Supplementary Figure 1: I-XIII Main fabrication steps of COC and TOC applied to graphene specimens. Schematic illustration of released symmetric and asymmetric crack-on-chip COC design, respectively, in XIV and XV. The inset shows the crack arrest length measured after the release step. This length will be used to determine the fracture toughness given by each COC structure. XVI tensile-on-chip TOC design with dogbone shape specimen. The red inset is a schematic zoomed view of a TOC showing the displacement measured between two cursors after the release step, one is fixed and one is movable. The displacement *u* is used in the analytical equation to determine the strain and stress provided by the TOC structure.

(a)

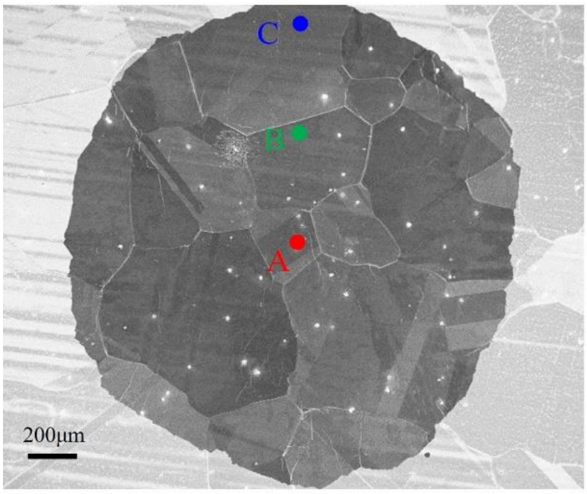

(b)

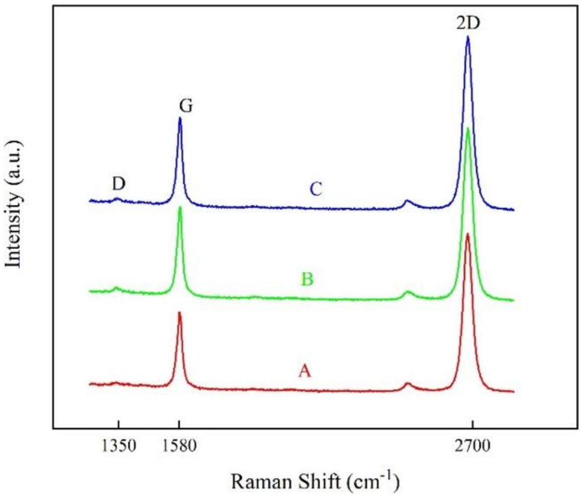

Supplementary Figure 2: Characterization of graphene domains by (a) SEM and (b) Raman (at 3 locations). G-band of around 1583 cm^{-1} is the typical value found for a flat graphene layer. The sharp 2D-band signal indicates a single-layer graphene.

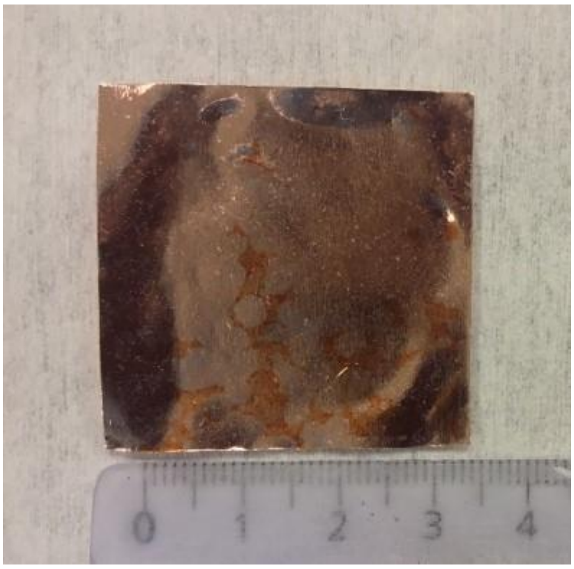

Supplementary Figure 3: The domain size of the tested CVD monolayer graphene is around 3 cm. The graphene layer covers a length of 3 cm of the Cu foil avoiding its oxidation.

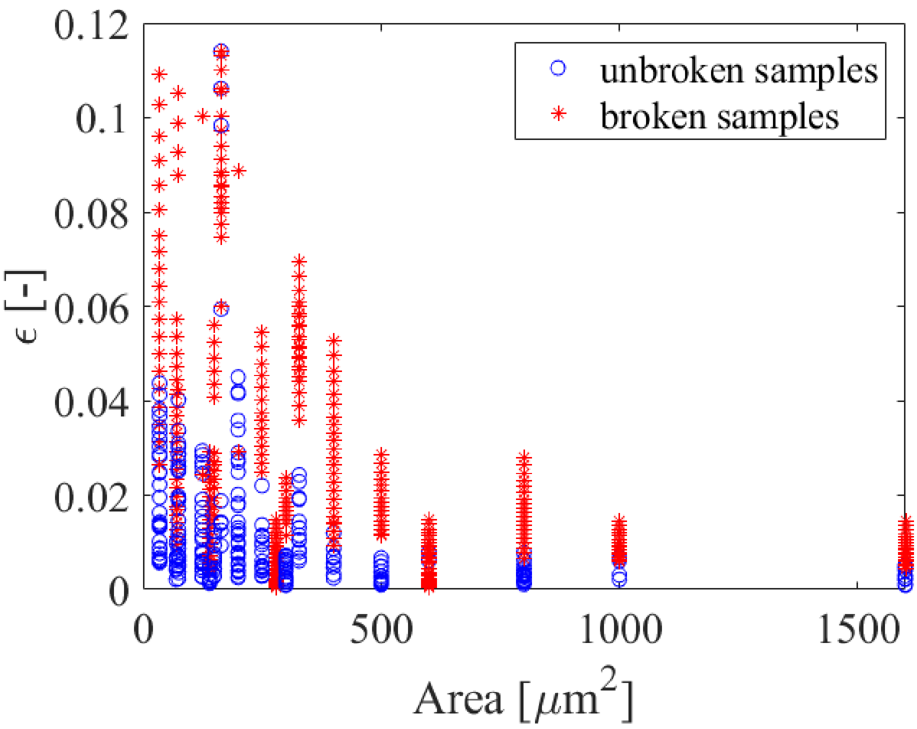

Supplementary Figure 4: TOC results of monolayer graphene as a function of the surface area of the graphene specimen. Red color for broken specimens and blue color for unbroken ones. The strain for an unbroken specimen is the measured one. The strain corresponding to a broken specimen is the maximum strain applied to this specimen, known based on equation S9.

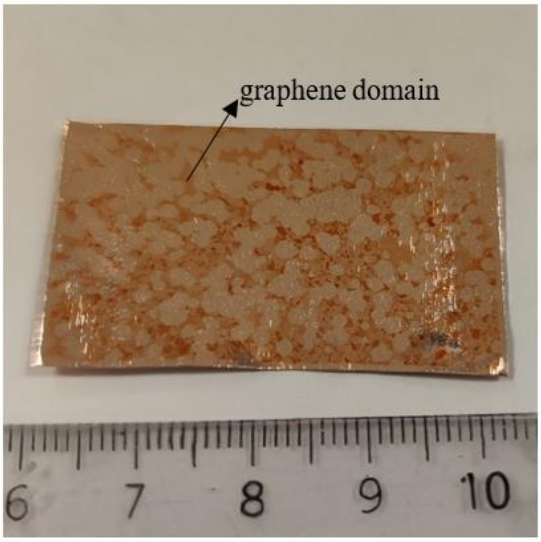

Supplementary Figure 5: Separated graphene domains on top of an oxidized Cu foil. The oxidation of copper foil occurred at the locations that were not covered by graphene (reddish zones).

Supplementary Note I: TOC mechanical model

The analytical equations describing the mechanics of the on-chip uniaxial tension test structures were developed based on linear elasticity as detailed in references [1-8]. The true stress and true strain are determined using the following relationships:

$$\varepsilon = \ln\left(\frac{L_s + u}{L_s}\right) - \varepsilon^{mis}, \quad \text{(S1)}$$

$$\sigma = A\left(\ln\left(\frac{L_a - u}{L_a}\right) - \varepsilon_a^{mis}\right)\exp(\varepsilon), \quad \text{(S2)}$$

where $A = E_a \frac{W_a t_a}{Wt}$. All parameters were determined experimentally with definition and values reported in Supplementary Table **1**. For the actuator, the mismatch strain can be determined using special test structures called self-actuated structures (shown in Supplementary Figure **6**) where the actuator beam pulls on a specimen made of the same material used as the actuator. The same technique can be used to determine the mismatch of the test specimen. However, such a test structure would not work for graphene. Alternatively, the mismatch strain can be determined using a free beam, which gives high accuracy when long enough. In practice, long free cantilevers even with a minor internal stress gradient over the thickness lead to significant out-of-plane displacement as shown in Supplementary Figure **7**. As out-of-plane displacement cannot be accurately measured, only short beams without any out-of-plane bending should be utilized, at the expense of accuracy (shorter beams involve shorter displacement upon release). In the present work, both techniques lead to a value close to the mismatch strain of the Ni actuator; $\varepsilon_a^{mis}\sim 0.003$ (self-actuated technique) and $\varepsilon_a^{mis}\sim 0.004$ (free beam). From one deposition to another, the actuator mismatch slightly changes due to several reasons related to deposition and specimen storing conditions. The latter parameter is measured in each release in order to take into account any slight variation.

Supplementary Table 1: Mechanical and geometrical parameters used in the FE simulations and uncertainties analysis for graphene specimens; E_a is Young's modulus of the actuator, E is Young's modulus of the specimen, σ_a^{int} is the internal stress of the actuator, σ^{int} is the internal stress of the specimen, ν_a is the Poisson's ratio of the actuator, ν is the Poisson's ratio of the specimen, t_a is the actuator thickness, t is specimen thickness, a is crack length, W_a is the actuator width, L_a the actuator length, L_s the specimen length, W the specimen width and W_a^s is the specimen overlap width.

**Parameter**	**Measurement method**	**Value**	**Uncertainty**
**Actuator Young's modulus (E_a)**	Nanoindentation	207 GPa	$\Delta E_a = 8$ GPa

**Specimen Young's modulus (E)**	Nanoindentation + AFM + literature[16,17]	~ 1 TPa	$\Delta E = 100$ GPa
**Internal stress of the actuator (σ_a^{int})**	Stoney measurement	600 MPa	$\Delta\sigma_a^{int} = 10$ MPa
**Internal stress of the specimen (σ^{int})**	Assumption	0 MPa	$\Delta\sigma^{int} = 10$ MPa
**Poisson's ratio of the actuator (ν_a)**	From literature[18]	0.3	$\Delta\nu_a$= 0.01
**Poisson's ratio of the specimen (ν)**	From literature[19-21]	~ 0.3	$\Delta\nu = 0.01$
**Thickness of actuator (t_a)**	Profilometry	70 nm	$\Delta t_a = 1$ nm
**Thickness of specimen (*t*)**	Raman & AFM	0.34 nm	$\Delta t = 0.02$ nm
**Crack length (*a*)**	SEM after release	Depending on each structure	$\Delta a = 50$ nm
**Actuator width (W_a)**	SEM before and after release	10.3 µm	$\Delta W_a = 50$ nm
**Actuator length (L_a)**	SEM (before & after)	Varies between 10 to 100 µm	$\Delta L_a = 100$ nm
**Specimen length (L_s)**	SEM (before & after)	6.0 - 7.7 µm	$\Delta L = 30$ nm
**Specimen width (*W*)**	SEM (before & after)	40 µm	$\Delta W = 30$ nm
**Specimen overlap width (W_a^s)**	SEM (before & after)	7.7 - 8.1 µm	$\Delta W_a^s = 30$ nm
**Notch radius**	SEM (before+after)	1.2 µm	$\Delta Radius = 40$ nm
**Notch length**	SEM (before+after)	Varies between 0 to 24 µm	$\Delta Length = 40$ nm

**Released width**	SEM after release using free beams (as shown in Supplementary Figure **8**)	Varies mostly between 10 to 14 µm	$\Delta L_{rel} = 500$ nm

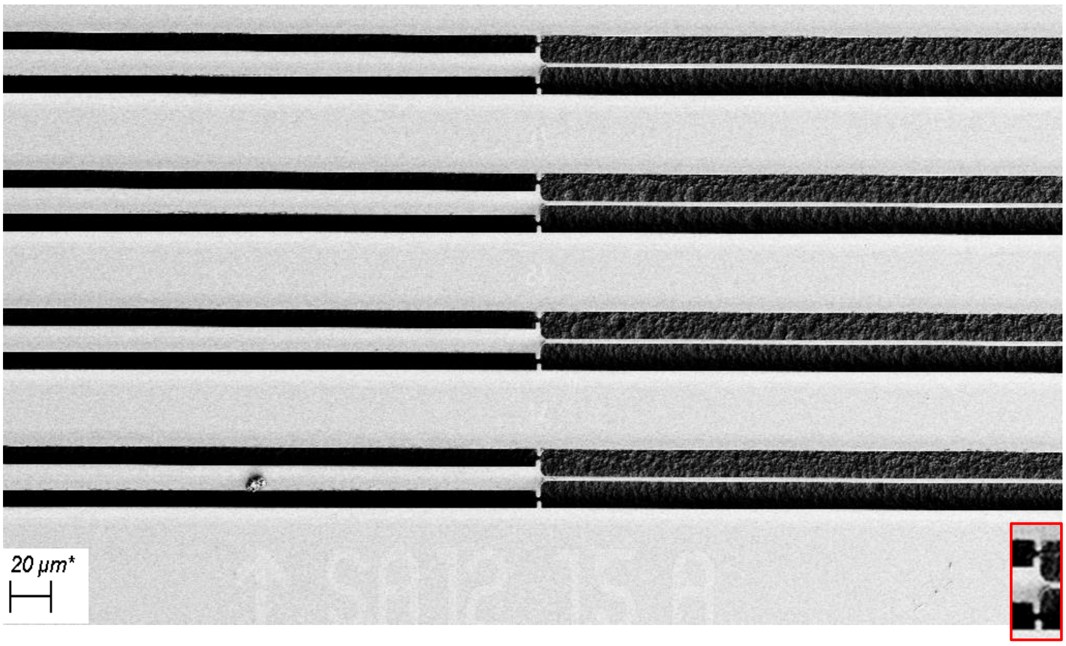

Supplementary Figure 6: SEM images of self-actuated structures of Ni films to determine the mismatch strain of the Ni actuator. Both beams are Ni films. Zoomed view of the displacement cursors in the inset.

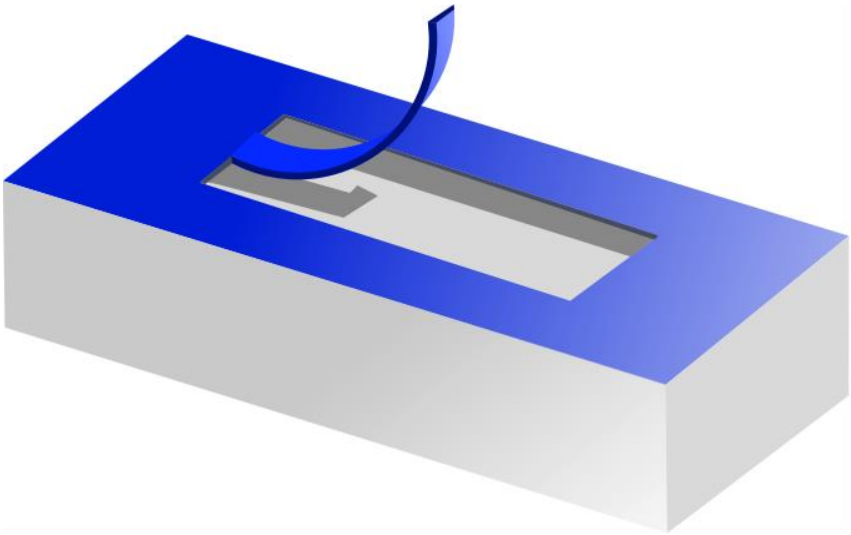

Supplementary Figure 7: Out-of-plane bending of a free actuator beam due to the internal stress gradient that is released prior to the etching of the underneath layer.

Supplementary Note II: Finite element simulation of COC

2D FE simulations have been performed using the commercial software Abaqus associated with Python scripts in order to establish an accurate data reduction scheme for the extraction of the mode I stress intensity K_I given by each structure as a function of the crack length and of the other geometrical dimensions. Details can be found in Jaddi *et al.*[9-10], and only the main aspects are summarized hereafter, in particular regarding the simulation of the release process. Furthermore, FE simulations were used to guide the design of the test structures and to analyze the kinetics of the release process.

A structure involving the test specimen, the two actuator beams, and a sufficiently wide zone of the surrounding substrate is meshed using 4-node bilinear plane stress elements with reduced integration (CPS4R). The collapsed quadrilateral quarter element technique is used to capture the singularity field near the crack tip. The overetching of the anchoring regions is taken into account as was performed in the previous study[10]. However, in this work, Ni was used as an actuator making the determination of the shape and size of the overetched zone not straightforward as opposed to the case where a transparent actuator. Thus, free beam structures (as shown in Supplementary Figure **8**) were used to determine the released area that was introduced in the simulation assuming the symmetry of the release. All nodes are initially fixed to represent the perfect adhesion to a rigid substrate. The linear isotropic elastic Hooke's law is invoked for both the specimen and actuator materials with, respectively, Poisson ratio, ν and ν_a, and Young's modulus, E and E_a.

At first, a fictitious thermal loading with the same magnitude as the measured equibiaxial internal stress was applied. Then the nodes were progressively released over time according to their shortest distance to one of the nodes located at the edges of the geometry. The node is released when the minimum calculated distance associated with a node is larger than the product of the etching rate and time. The release is stopped when the final etching time is attained, leaving several nodes at the periphery of the model unreleased, hence dictating the clamping frontier. Consequently, the value is directly provided in the form of J-integral values.

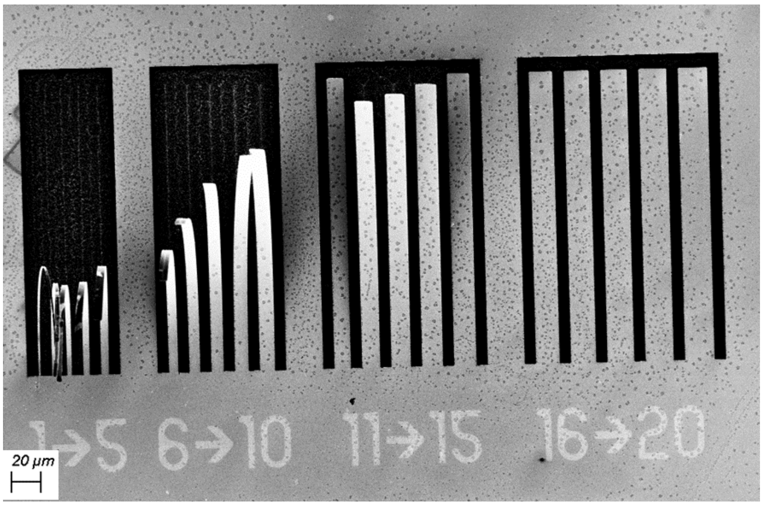

Supplementary Figure 8: SEM micrograph showing free beams with different widths used to measure the released width thus determining the overetch width. Here the 14 µm-wide beam shows out-of-plane bending which implies a 7 µm underetch. These structures are used when it is difficult to see the release profile especially when non-transparent material is used as an actuator like Ni film.

Supplementary Note III: COC principle

For short crack length a, the stress intensity factor can be approximately expressed by[9]

$$K_{I\,asym/sym} = \left(1-\nu_a\right)\sigma_a^{\text{int}}\sqrt{L_a}\,\frac{Y\sqrt{\pi\frac{a}{W}}\sqrt{\frac{W}{L_s}}\sqrt{\frac{L_a}{L_s}}}{\frac{L_a}{L_s}\frac{t}{t_a}+\frac{E_a}{2E}\left(\alpha_2 Y^2\pi\left(\frac{a}{W}\right)^2\frac{W}{L_s}+\alpha_3\right)}, \quad \text{(S3)}$$

with $Y = 1.12$ or 1 in the case of asymmetric geometry or symmetric, respectively. The two actuator beams have a length L_a, width W_a (or $2W_a$ for the symmetric design), thickness t_a, internal stress σ_a^{int}, Young's modulus E_a, and Poisson ratio ν_a. The test specimen has a length L_s, width W ($2W$ for the symmetric design), thickness t, internal stress σ^{int} that is considered equal to zero in the case of graphene, Young's modulus E, Poisson ratio ν, $\alpha_2 = 1-\nu^2$ in plane strain and $\alpha_2 = 1$ in plane stress, and $\alpha_3 = 1-\nu^2$ when the specimen is attached at its upper and lower edges and $\alpha_3 = 1$ when the specimen is free at the edges and involves a crack of length a ($2a$ for the symmetric design).

For long crack length a, the expression is[9]

$$K_{I\;DCB\,asym} = \left(1-\nu_a\right)\sigma_a^{\text{int}}\sqrt{L_a}\,\frac{4\sqrt{\frac{6L_a}{\alpha_2 L_s}}}{32\frac{E_a}{E}\frac{a^2}{L_s^2}+\frac{L_s}{a}\frac{L_a}{W_a}\frac{t}{t_a}}, \quad \text{(S4)}$$

$$K_{I\;DCB\,sym} = \left(1-\nu_a\right)\sigma_a^{\text{int}}\sqrt{L_a}\,\frac{4\sqrt{\frac{6L_a}{\alpha_2 L_s}}}{32\frac{E_a}{E}\frac{a^2}{L_s^2}+\frac{L_s}{a}\frac{L_a}{W_a}\frac{t}{t_a}}, \quad \text{(S5)}$$

For the graphene case, the ratio $\frac{t}{t_a}$ is almost zero. Therefore, the above equations can be simplified as for short crack length a:

$$K_{I\,asym/sym} = \left(1-\nu_a\right)\sigma_a^{\text{int}}\sqrt{L_a}\,\frac{Y\sqrt{\pi\frac{a}{W}}\sqrt{\frac{W}{L_s}}\sqrt{\frac{L_a}{L_s}}}{\frac{E_a}{2E}\left(\alpha_2 Y^2\pi\left(\frac{a}{W}\right)^2\frac{W}{L_s}+\alpha_3\right)}, \quad \text{(S6)}$$

and for long crack length a:

$$K_{I\,DCB\,asym/sym} = \left(1-\nu_a\right)\sigma_a^{\mathrm{int}}\sqrt{L_a}\,\frac{EL_s^2\sqrt{\dfrac{6L_a}{\alpha_2 L_s}}}{8E_a a^2}, \tag{S7}$$

Supplementary Note IV: Unsuccessful COC tests

Supplementary Figure **9** shows examples of specimen breaks before any crack initiation at the notch tip. Supplementary Figure **9**(a) shows the fracture case that often occurs in symmetric configurations and which consists of the failure of the graphene specimen in the middle part from both attached sides to both actuators or one side as shown in Supplementary Figure **9**(b). While sometimes the specimen breaks in the anchored beam from either one side or both sides as in Supplementary Figure 9(d) and (c), respectively. This fracture was driven by the fact that the two long parts of the specimen twisted due to the long length and the thinness of the graphene. Therefore, for future works, in order to be able to produce more reliable and successful symmetric structures for 2D materials, some design modifications appear necessary. A possible improvement of the symmetric design can be the large reduction of the specimen length or the addition of a new layer on top of graphene where the fracture likely occurred and far away from the cracking path. Another problem that occurred during the release is the specimen's edges twisting, especially the notch edges where the twisting can be very large as shown in Supplementary Figure **9**(e), and can transform the specimen almost into a nanotube. This problem is also associated with out-of-plane displacement and is more acute in the case of long notches. Besides the problems related to specimen design, many COC devices were not working due to many problems encountered during the fabrication process. The most common fabrication problems are the resist residues and Ni redepositing as illustrated in Supplementary Figure **9**(f).

As mentioned previously the release is performed using XeF_2 which causes fluorination if the exposure time is long introducing some defects that could affect the graphene's mechanical properties[11]. However, in our case the exposure time was very short, in total it did not exceed 40 s. Moreover, some of the samples were re-measured months after the release to check the fluorination effects since the fluorination percentage has the tendency to decrease from 50% to 80% in the week that follows the exposition[12]. Therefore, we believe that the graphene used here is not fluorinated or the fluorination effects are minuscule.

The errors generated during the crack length measurement are likely produced due to, (i) the electron charging effect that causes further cracking which is not taken into account in the extracted K_{Ic}. Although the magnification is fixed and chosen in a way to not produce further cracking, still some structures can show more charging than others; (ii) wrapping or out-of-plane bending that is more critical in graphene structures making an accurate measurement of the crack length almost impossible.

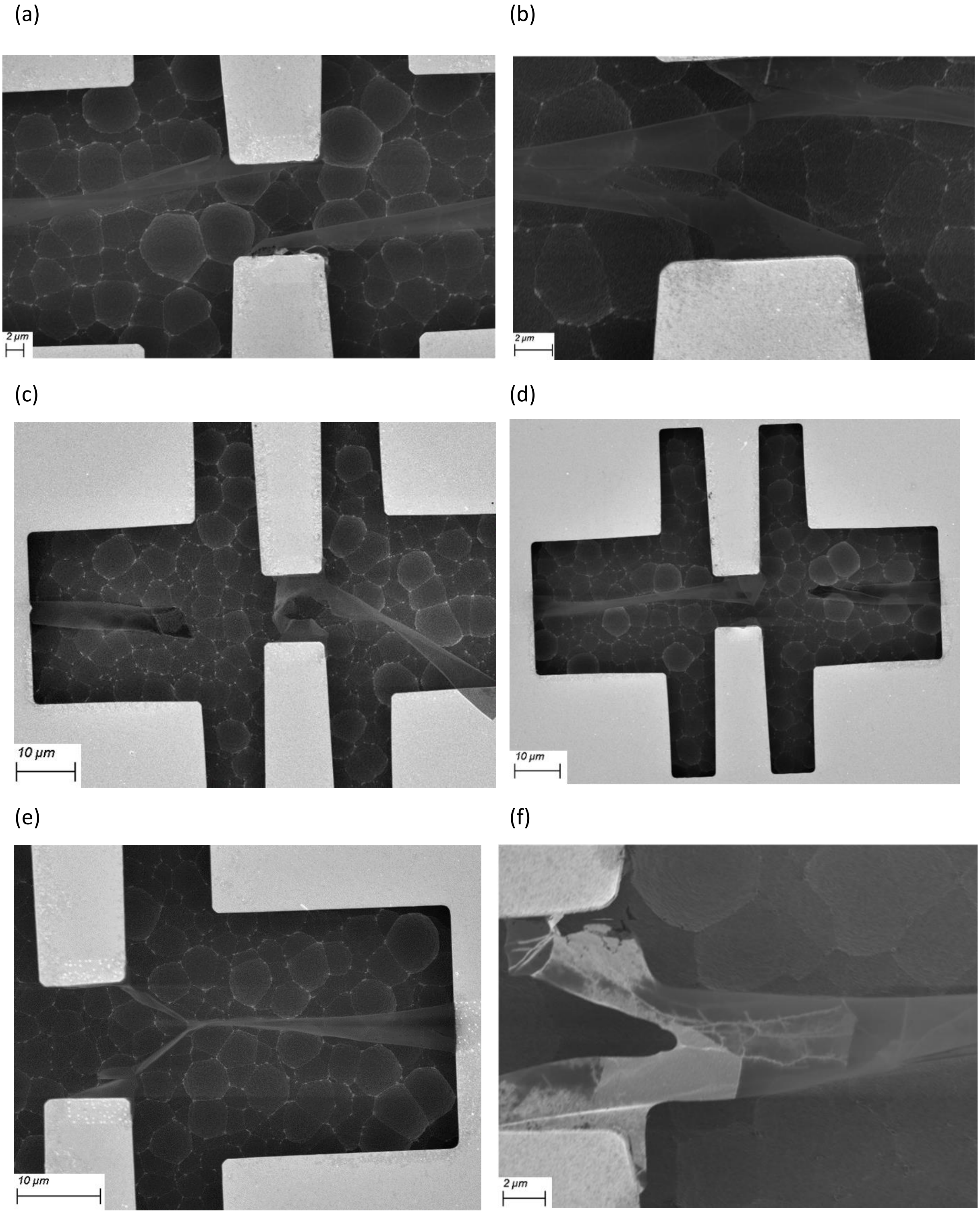

Supplementary Figure 9: Main problems encountered while testing crack-on-chip COC specimens; (a) fracture occurred in the part between the actuator on both sides, up and down; (b) fracture occurred far from the notch in the clamped beam of the graphene; (c) same as (b) but with another fracture of the other specimen's beam near the overlap between the graphene and the substrate; (d) fracture of the overlap and the specimen's beam on one side; (e) twisting of the specimen edges, precisely the notch ones; (f) resist residues and redepositing of Ni film on top of graphene membrane. The structures that undergo these problems are discarded.

Supplementary Note V: Error propagation on *K* in the case of graphene

In order to perform the error analysis, we will follow the same steps performed previously for SiO_2 in[10]. The need to redo this error propagation is necessary since here the specimen thickness is negligible and Young's modulus is very high. Therefore, the assumptions which were adopted before to simplify the analysis are no longer valid.

We rely on the analytical formula (S7). The expression includes the following independent variables: Poisson's ratio of the actuator ν_a, internal stress in the actuator σ_a^{int}, length of the actuator L_a, Young's modulus of the actuator E_a, Young's modulus of the specimen E, crack length a, the thickness of the actuator t_a, thickness of the specimen t, length of the specimen L_s, the width of the actuator W_a, $\alpha_2 = 1 - \nu^2$ where ν is the Poisson's ratio of the specimen.

We neglect the errors on W_a, t and t_a which are very small, less than 0.001%. The ratio $\frac{t}{t_a}$ tends to zero hence, the second term of the denominator is neglected in the following analysis. The formula for determining the relative error on *K* from the propagation of the different uncertainties is

$$\frac{\Delta K}{K} = \sum_i \left|\frac{\partial K}{\partial x_i}\right| \frac{\Delta x_i}{K}, \tag{S8}$$

where x_i refers to the different independent variables listed above.

The eight contributions to the overall uncertainty are derived one by one. The three first contributions from σ_a^{int}, ν_a and α_2 lead to systematic constant errors

$$\left|\frac{\partial K}{\partial \sigma_a^{int}}\right| \frac{\Delta \sigma_a^{int}}{K} = \frac{\Delta \sigma_a^{int}}{\sigma_a^{int}} = 1.67\%,$$

$$\left|\frac{\partial K}{\partial \nu_a}\right| \frac{\Delta \nu_a}{K} = \frac{\Delta \nu_a}{1 - \nu_a} = 1.4\%,$$

$$\left|\frac{\partial K}{\partial \alpha_2}\right| \frac{\Delta \alpha_2}{K} = \frac{\Delta \alpha_2}{2} = 0.55\%.$$

Since the second term of the denominator in equation (S7) is neglected, the uncertainty on E, E_a and on the specimen length lead to a constant contribution to the overall error:

$$\left|\frac{\partial K}{\partial E_a}\right| \frac{\Delta E_a}{K} = \frac{\Delta E_a}{E_a} = 3.86\%,$$

$$\left|\frac{\partial K}{\partial E}\right| \frac{\Delta E}{K} = \frac{\Delta E}{E} = 10\%,$$

$$\left|\frac{\partial K}{\partial L_s}\right|\frac{\Delta L_s}{K}=\frac{3}{2}\frac{\Delta L_s}{L_s}=0.58\%.$$

The following errors vary from one structure to another:

$$\left|\frac{\partial K}{\partial L_a}\right|\frac{\Delta L_a}{K}=\frac{\Delta L_a}{L_a}<0.9\%,$$

$$\left|\frac{\partial K}{\partial a}\right|\frac{\Delta a}{K}=\frac{2\Delta a}{a}<3.2\%.$$

The dominant contribution is the error coming from the determination of Young's modulus of the graphene specimen.

In summary, the sum of errors is, in the best case, around 17% and can increase up to 23% in the case of short crack lengths and short actuator lengths. However, considering several specimens reduces the error on the determined mean fracture toughness.

Other phenomena might also affect the accuracy of the extracted fracture toughness that is not accounted for in the above uncertainty analysis. For instance, some values at the limit of the distribution are associated with specimens exhibiting wrinkles near the notch tip, which are known to weaken the specimen and accelerate failure[13], although, in other studies, wrinkles are considered as offering extra resistance to crack propagation[14]. In any case, wrinkles artificially modify the fracture toughness, an effect not accounted for in our uncertainty analysis. Moreover, thick graphene islands were revealed to react as crack arrestors preventing sudden crack propagation[14]. Resist residues can also act as crack arrestors and affect further the cracking rate compared with the fracture toughness. All these phenomena are described in more details in the Supplementary Material VI.

Supplementary Note VI: On the sources of imperfection/pollution in the graphene on-chip tests

In this section, the possible factors that could alter the quality of the graphene specimens are analyzed such as the presence of corrugations and PMMA residues. It also discusses how the mechanical properties are potentially influenced by the corrugations or the resist residues.

2D materials unlike their 1D or 0D counterparts exhibit corrugations such as wrinkles, ripples, creases, or crumples. These corrugations can be categorized based on their aspect ratio, order, and topology. More information regarding these corrugations has been collected in the review paper by Deng *et al*[22]. Wrinkles typically have a length longer than 100 nm, a height below 10 nm, and a high aspect ratio with widths between 1 to tens of nm. Ripples have an aspect ratio of around 1nm. Both are temporary distortions that disappear once the responsible loading conditions like compressive buckling are removed or a tensile load is applied to the sample. On the other hand, creases constitute a kind of permanent folds that are generated by applying high plastic deformation leading to a shorter sample compared with its original initial in-plane length. Last, crumples can be considered as intense wrinkles and folds occurring mostly with a 3-dimensional topology similar to a crumpled paper.

Numerous reasons lead to these corrugations in graphene such as dislocations, surface anchorage, substrate relaxation, edge or interatomic interaction instabilities, and surface tension caused by solvents. For instance, the wrinkles are known to be denser near the graphene edges and defects due to the higher asymmetric distribution of bond lengths.[23,24] Wrinkles/ripples are dependent mainly on the surface morphology of the growth substrate and on the transfer process.

Literature shows that the surface morphology underneath the graphene controls the wrinkles' density as well as their orientation.[25] Moreover, the roughness of the surface also controls the wrinkles' density since high roughness introduces extra stress to ensure adhesion between the layers leading to an increase in the number of wrinkles. Another important point is the difference in the thermal coefficient between the graphene and the underlying layer that can generate wrinkles. For instance, the epitaxy growth of graphene on top of SiC wafers shows higher wrinkles during cooling due to thermal expansion. [26,27]

In most cases, graphene is grown on top of metallic surfaces requiring to be transferred to another substrate, most likely Si, thus tending to form wrinkles. The latter can hardly be suppressed, except with very specific fine-tuned methods like the one developed by Chatterjee *et al.*[28] This transfer step could not be avoided in this study as well. Regardless of the great care taken during the graphene transfer step, we indeed still see some wrinkles in our specimens. Nevertheless, the graphene tested in this work has a low density of wrinkles thanks to the transfer method based on soaking PMMA/graphene layer in deionized water at a high temperature of around 80°C and making sure the Si surface is hydrophobic. The study conducted by Liu *et al.*[29] also by Gao *et al.*[30] shows the advantage of this transfer technique. A key point is that the area where the test structures were patterned in this work was carefully selected in a region with no or very few amount of wrinkles.

Nevertheless, the presence of wrinkles is minimized in this work especially when designing the test specimens, the etching of the underlayer also re-introduces wrinkles into graphene that becomes unstable, tending to self-folding especially with wide samples as observed in this work, and in other reported studies, e.g. in Lambin *et al*.[31] and Cranford *et al*.[32] These out-of-plane ripples/wrinkles manifest in freestanding graphene as a way to release the in-plane strain energy. The wrinkle wavelength and density increase under an applied strain especially near the edges of wide samples.

Consequently, the graphene used in this work does have some corrugations mostly in the form of wrinkles or creases. Thus, the core question is the impact of the presence of corrugations on the extracted properties. The influence of corrugations on the extracted mechanical properties is not a trivial question. It is very challenging to quantify, and up to now, to our knowledge, there is no work studying this impact experimentally in detail. To explore the potential implications, however, some research based on numerical models and theoretical calculations was carried out. According to Xi Shen *et al*.'s[33] research wrinkles have typically very little effect on the monolayer graphene sheet. For example, Young's modulus along the armchair direction is reduced by around 11% for high wrinkle density with high wavelength. The measurement error on our estimated Young's modulus, 0.85 TPa and 1.2 TPa (resulting from dogbone and rectangular samples, respectively), is in the region of 4% reduction along the zigzag direction. On the other hand, certain research, such as that by Qin *et al.*[34] demonstrated greater strength for wrinkled graphene compared to flat graphene.

Thanks to its high strength, wrinkled graphene was employed by Zhao *et al*.[35,36] to reinforce various metal matrix composites. According to Akhunova et al.'s research,[37] contrary to high-amplitude corrugations, small-amplitude corrugations in graphene do not significantly lower its Young's modulus and fracture strength. Only small-amplitude corrugations are expected to marginally lower the calculated Young's modulus and fracture strength in comparison to the pristine values. Additionally, a negative Poisson ratio was demonstrated. [34] In essence, the density of corrugations in the used samples of the present work is low and thus has a very minor impact on the extracted Young’s modulus, strength, and fracture strain; this is consolidated by the repeatability of the results coming from different samples, although the non-uniformity of the corrugations distributions, and by the fact that the obtained properties values are similar to the reported values in the literature.

Regarding fracture toughness, wrinkles alter the extracted value of fracture toughness in an artificial way that is not taken into consideration by our uncertainty analysis. It is likely that some values at the limit of the distribution are related to specimens with wrinkles near the notch tip, which are known to weaken the sample and speed up failure, [38] despite the fact that in some studies wrinkles are thought to provide an additional barrier against crack propagation.[39] Nevertheless, the probability of the presence of wrinkles in the vicinity of the notch tip is negligible, especially in all the test 80 samples.

On the other hand, creases have been observed more in the asymmetric as a result of imperfections and misalignments in the geometry that can introduce significant out-of-plane displacement and can be considered somehow responsible for the discrepancy of the obtained fracture toughness values. While

the TOC (uniaxial specimens) and symmetric COC specimens do not exhibit substantial creasing upon deformation due to more constraints placed on the test structure, it is possible that the creases are encouraged by the existence of the crack. We have been running 3D FE simulations with Abaqus with imperfections, but not with the goal to generate creases and study them, but to estimate the mixed mode effects on cracking. The analysis was not pushed further because the effects were found weak. These simulations were further pursued by Twente's team who delivered convincing data. [40] The fact is that these 3D simulations never lead to creasing. This is often the case with such types of instabilities that one needs to seed the right imperfection to generate it. This appears to be a research project on its own that goes beyond what can be achieved in the context of this study.

The literature has demonstrated that PMMA-based graphene transfer results in either some form of PMMA islands or a continuous layer of residues with a thickness of 1 to 2 nm. Consequently, even in very minute amounts, PMMA residues can be found in some places of graphene. After every transfer of graphene, an SEM analysis is performed to ensure that there are no residues, at least not of a size that can be detected, in the area of interest. We are unable to offer conclusive reasoning to exclude very tiny residues. The only thing we can conclude is that the removal of PMMA residues is aided by the combination of annealing and hot acetone as was demonstrated by Hwangbo *et al.*[41] Here, the presence of a continuous film of PMMA is unlikely but we have some islands of residues with not high density thanks to using hot acetone. Hwangbo *et al.*[41] demonstrate that the bonding strength between graphene and the PMMA residues is very weak. Therefore, synergetic toughness enhancement of the graphene and residues even in the case of a uniform layer of PMMA is unlikely. In our case, the fracture toughness will not be impacted by the presence of some small/thin islands of residues, especially since the fracture toughness of PMMA is low around 1 MPa$\sqrt{m}$. However, in general, the cracking rate and path can be influenced in the case of large/thick residues that can behave as crack arrestors.

Now, assuming these minor residues would in any case be attached to certain specimens, one can reasonably expect that they would not significantly affect the mechanical behavior of graphene. This is likewise the interest of performing a large number of tests to limit the impact of some defective specimens.

As a final remark, it is worth mentioning that the step that introduces more PMMA residues is not the graphene transfer but the lithography step. This step used PMMA underneath the photoresist since residues of PMMA are easier to remove than photoresist. The removal of PMMA in this step is based on hot acetone rinses leaving a layer of resist residues. Moreover, characterizing graphene with PMMA residues is still interesting since the fabrication of graphene-based transistors and logic circuits strongly relies on PMMA as well.

As a conclusion, both PMMA residues and the different forms of corrugations can slightly change the obtained mechanical properties of the monolayer graphene.

Supplementary Note VII: When is the measured graphene displacement significantly smaller than u_{free}?

The comparison between the displacement measured when the graphene is present and with a free beam having the same length leads to a different value, which proves that although a very thin graphene layer induces a significant stress in the actuator that leads to a measurable effect on displacement. This can also be checked analytically by:

$$u = \frac{1}{1 + \frac{EWtL_{a0}}{E_a W_a t_a L_{s0}}} u_{free}. \tag{S9}$$

The second term of the denominator in equation (S9) increases when the ratio $\frac{WL_{a0}}{W_a L_{s0}}$ increases. By replacing the parameters in equation (S9) with the corresponding values. For instance, for the shortest actuator of a structure 'Rect', $u = 0.97u_{free}$, while for the longest actuator $u = 0.4u_{free}$. Therefore, for short actuator lengths, the actuator reacts like a free beam while for longer beams the difference between u and u_{free} is significant and cannot be neglected. Consequently, an approximate estimation of the stress in the monolayer graphene specimen can be determined using the TOC technique, which allows building a stress-strain response.

Supplementary References

1. Fabregue, D., Andre, N., Coulombier, M., Raskin, J. P., & Pardoen, T. Multipurpose nanomechanical testing machines revealing the size-dependent strength and high ductility of pure aluminium submicron films. *Micro & Nano Letters*, **2**, 13-16 (2007).
2. Gravier, S. et al. New on-chip nanomechanical testing laboratory-applications to aluminum and polysilicon thin films. *Journal of Microelectromechanical Systems*, **18**, 555-569 (2009).
3. Coulombier, M., Boe, A., Brugger, C., Raskin, J. P., & Pardoen, T. Imperfection-sensitive ductility of aluminium thin films. *Scripta Materialia*, **62**, 742-745 (2010).
4. Coulombier, M. et al. On-chip stress relaxation testing method for freestanding thin film materials. *Review of Scientific Instruments*, **83**, 105004 (2012).
5. Bhaskar, U. et al. On-chip tensile testing of nanoscale silicon free-standing beams. *Journal of Materials Research*, **27**, 571-579 (2012).
6. Colla, M. S. et al. Dislocation-mediated relaxation in nanograined columnar palladium films revealed by on-chip time-resolved HRTEM testing. *Nature Communications*, **6**, 1-8 (2015).

7. Vayrette, R., Raskin, J. P., & Pardoen, T. On-chip fracture testing of freestanding nanoscale materials. *Engineering Fracture Mechanics*, **150**, 222-238 (2015).
8. Ghidelli, M. et al. Homogeneous flow and size dependent mechanical behavior in highly ductile Zr65Ni35 metallic glass films. *Acta Materialia*, **131**, 246-259 (2017).
9. Jaddi, S., Coulombier, M., Raskin, J. P., and Pardoen, T. Crack on a chip test method for thin freestanding films. *Journal of the Mechanics and Physics of Solids*, **123**, 267-291 (2019).
10. Jaddi, S., Raskin, J. P., & Pardoen, T. On-chip environmentally assisted cracking in thin freestanding SiO2 films. *Journal of Materials Research*, **36**, 1-16 (2021).
11. Davami, K. *et al*. Modification of mechanical properties of vertical graphene sheets via fluorination. *RSC advances,* **6**, 11161-11166 (2016).
12. Stine, R., Lee, W. K., Whitener Jr, K. E., Robinson, J. T., and Sheehan, P. E. Chemical stability of graphene fluoride produced by exposure to XeF2. *Nano letters*, **13**, 4311-4316 (2013).
13. Wang, L., Williams, C. M., Boutilier, M. S., Kidambi, P. R., & Karnik, R. Single-layer graphene membranes withstand ultrahigh applied pressure. *Nano Letters*, **17**, 3081-3088 (2017).
14. Hwangbo, Y. et al. Fracture characteristics of monolayer CVD-graphene. *Scientific Reports*, **4**, 1-9 (2014).
15. Laconte, J. et al. Thin films stress extraction using micromachined structures and wafer curvature measurements. *Microelectronic Engineering,* **76**, 219-226 (2004).
16. Lee, C., Wei, X., Kysar, J. W., & Hone, J. Measurement of the elastic properties and intrinsic strength of monolayer graphene. *Science,* **321**, 385-388 (2008).
17. Zhang, Y., & Pan, C. Measurements of mechanical properties and number of layers of graphene from nano-indentation. *Diamond and Related Materials*, **24**, 1-5 (2012).
18. Wang, Z., Ma, Z., Zhou, Y., & Lu, C. Measurement of the mechanical properties of nickel film based on the full-field deformation: An improved blister method. *Progress in Natural Science: Materials International*, **23**, 453-458 (2013).
19. Lu, J. P. Elastic properties of carbon nanotubes and nanoropes. *Physical Review Letters,* **79**, 1297 (1997).
20. Sammalkorpi, M., Krasheninnikov, A., Kuronen, A., Nordlund, K., & Kaski, K. Mechanical properties of carbon nanotubes with vacancies and related defects. *Physical Review B,* **70**, 245416 (2004).
21. Cadelano, E., Palla, P. L., Giordano, S., & Colombo, L. Nonlinear elasticity of monolayer graphene. *Physical Review Letters,* **102**, 235502 (2009).
22. Deng, S., & Berry, V. Wrinkled, rippled and crumpled graphene: an overview of formation mechanism, electronic properties, and applications. *Materials Today,* **19**, 197-212 (2016).
23. Shenoy, V. B., Reddy, C. D., Ramasubramaniam, A., & Zhang, Y. W. Edge-stress-induced warping of graphene sheets and nanoribbons. *Physical review letters*, **101**, 245501 (2008).

24. Fasolino, A., Los, J. H., & Katsnelson, M. I. Intrinsic ripples in graphene. *Nature Materials,* **6**, 858-861 (2007).
25. Calado, V. E., Schneider, G. F., Theulings, A. M. M. G., Dekker, C., & Vandersypen, L. M. K. Formation and control of wrinkles in graphene by the wedging transfer method. *Applied Physics Letters*, **101**, (2012).
26. Vecchio, C., Sonde, S., Bongiorno, C., Rambach, M., Yakimova, R., Raineri, V., & Giannazzo, F. Nanoscale structural characterization of epitaxial graphene grown on off-axis 4H-SiC (0001). *Nanoscale Research Letters,* **6**, 1-7 (2011).
27. Biedermann, L. B., Bolen, M. L., Capano, M. A., Zemlyanov, D., & Reifenberger, R. G. Insights into few-layer epitaxial graphene growth on 4 H-SiC (000 1) substrates from STM studies. *Physical Review B*, **79**, 125411 (2009).
28. Chatterjee, S., Kim, N. Y., Pugno, N. M., Biswal, M., Cunning, B. V., Goo, M., ... & Ruoff, R. S. Synthesis of highly oriented graphite films with a low wrinkle density and near-millimeter-scale lateral grains. *Chemistry of Materials*, **32**, 3134-3143 (2020).
29. Liu, N., Pan, Z., Fu, L., Zhang, C., Dai, B., & Liu, Z. The origin of wrinkles on transferred graphene. *Nano Research*, **4**, 996-1004 (2011).
30. Gao, L., Ni, G. X., Liu, Y., Liu, B., Castro Neto, A. H., & Loh, K. P. Face-to-face transfer of wafer-scale graphene films. *Nature*, **505**, 190-194 (2014).
31. Lambin, P. Elastic properties and stability of physisorbed graphene. *Applied Sciences*, **4**, 282-304 (2014).
32. Cranford, S., Sen, D., & Buehler, M. J. Meso-origami: folding multilayer graphene sheets. *Applied physics letters*, **95**, (2009).
33. Shen, X., Jia, J., Chen, C., Li, Y., & Kim, J. K. Enhancement of mechanical properties of natural fiber composites via carbon nanotube addition. *Journal of materials science*, **49**, 3225-3233 (2014).
34. Qin, H., Sun, Y., Liu, J. Z., Li, M., & Liu, Y. Negative Poisson's ratio in rippled graphene. *Nanoscale*, **9**, 4135-4142 (2017).
35. Zhao, S., Zhang, Y., Yang, J., & Kitipornchai, S. Improving interfacial shear strength between graphene sheets by strain-induced wrinkles. *Carbon*, **168**, 135-143 (2020).
36. Zhao, S., Zhang, Y., Yang, J., & Kitipornchai, S. Folded graphene reinforced nanocomposites with superior strength and toughness: A molecular dynamics study. *Materials Science & Technology*, **120,** 196-204 (2022).
37. Akhunova, A. K., Galiakhmetova, L. K., & Baimova, J. A. The Effects of Dislocation Dipoles on the Failure Strength of Wrinkled Graphene from Atomistic Simulation. A*pplied Sciences*, **13**, 9 (2022).

38. Wang, L., Williams, C. M., Boutilier, M. S., Kidambi, P. R., & Karnik, R. Single-layer graphene membranes withstand ultrahigh applied pressure. *Nano Letters,* **17**, 3081-3088 (2017).
39. Hwangbo, Y. et al. Fracture characteristics of monolayer CVD-graphene. *Scientific Reports,* **4**, 1-9 (2014).
40. Shafikov, A., van de Kruijs, R., Benschop, J., Houweling, S., & Bijkerk, F. Fracture toughness of freestanding $ZrSi_x$ thin films measured using crack-on-a-chip method. *Journal of Microelectromechanical Systems,* **31**, 63-73 (2021).
41. Hwangbo, Y. et al. Fracture characteristics of monolayer CVD-graphene. *Scientific Reports,* **4**, 1-9 (2014).
