# Supplementary material for: Definitive engineering strength and fracture toughness of graphene through on-chip nanomechanics
Source: Nat Commun. 2024 Jul 12;15:5863. doi: 10.1038/s41467-024-49426-3 (PMC11245622; doi:10.1038/s41467-024-49426-3)
Supplement: Supplementary file 3 — Description of Additional Supplementary Files [file 41467_2024_49426_MOESM3_ESM.pdf]

Description of Additional Supplementary Files

File Name: Supplementary Code

Description: This file is the Python code that was used to calculate the fracture toughness in this paper based on the finite element model.
